# FTO promotes liver inflammation by suppressing m6A mRNA methylation of IL-17RA

**DOI:** 10.3389/fonc.2022.989353

**Published:** 2022-09-12

**Authors:** Xiaojie Gan, Zhihui Dai, Chunmei Ge, Haozan Yin, Yuefan Wang, Jian Tan, Shuhan Sun, Weiping Zhou, Shengxian Yuan, Fu Yang

**Affiliations:** ^1^ The department of Medical Genetics, Naval Medical University, Shanghai, China; ^2^ The Third Department of Hepatic Surgery, Eastern Hepatobiliary Surgery Hospital, Naval Medical University, Shanghai, China

**Keywords:** fto, IL-17RA, RNA methylation, m6A, liver inflammation, HCC

## Abstract

**Background:**

Previous studies have demonstrated that inflammation-related interleukin-17 (IL-17) signaling plays a pivotal role in the pathogenesis of non-alcoholic steatohepatitis (NASH)- and alcoholic liver disease (ALD)-induced hepatocellular carcinoma (HCC). However, rare efforts have been intended at implementing the analysis of N6-methyladenosine (m6A) mRNA methylation to elucidate the underpinning function of the IL-17 receptor A (IL-17RA) during the inflammation-carcinogenesis transformation of HCC.

**Methods:**

We performed methylated RNA immunoprecipitation sequencing (MeRIP-seq) using normal, HCC tumor and paired tumor adjacent tissues from patients to investigate the dynamic changes of m6A mRNA methylation in the process of HCC. Additionally, murine non-alcoholic fatty liver disease (NAFLD) model and murine chronic liver injury model were utilized to investigate the role of IL-17RA regulated by m6A mRNA modulator fat mass and obesity-associated (FTO) in chronic hepatic inflammation.

**Results:**

MeRIP-seq revealed the reduction of m6A mRNA methylation of IL-17RA in tumor adjacent tissues with chronic inflammation, suggesting the potential role of IL-17RA in the inflammation-carcinogenesis transformation of HCC. Besides, we demonstrated that FTO, rather than methyltransferase-like 3 (METTL3), methyltransferase-like 14 (METTL14), and alkB homolog 5 (ALKBH5) functions as a main modulator for the decrease of m6A mRNA methylation of IL-17RA *via* knockdown and overexpression of FTO *in vitro* and *in vivo*.

**Conclusions:**

Overall, we elaborated the underlying mechanisms of the increase of IL-17RA resulting in chronic inflammation *via* the demethylation of FTO in tumor adjacent tissues and demonstrated that targeting the specific m6A modulator FTO may provide an effective treatment for hepatitis patients to prevent the development of HCC.

## Introduction

Chronic inflammation is closely related to the initiation and progression of the tumor diseases, and about a quarter of cancers benefit from inflammatory infections during their development (1, 2). In 1863, Virchow firstly observed leukocytes in neoplastic tissues (3). Subsequently, accumulating studies reported that inflammation plays an important role in tumorigenesis and can increase the risk of cancer (4–6). It is a well-established fact that hepatocellular carcinoma (HCC) is still the most common cause of cancer-related mortality worldwide and arises from chronic hepatic inflammation (7, 8). The prevalence of chronic liver inflammation is increasing and becoming a major risk factor for the development of HCC (9). Therefore, a better understanding of the mechanisms leading to chronic hepatic inflammation is urgently needed for employing novel therapeutic strategy to strengthen the prevention of HCC.

N6-methyladenosine (m6A) modification is the most frequent form of internal RNA modification in eukaryotic cells and regulates almost every step of RNA metabolism, such as mRNA degradation, translation and splicing to control post-transcriptional gene expression in a large body of biological processes (10). m6A modification in mRNA is a dynamic and reversible modification, which is introduced by m6A methyltransferase, aka ‘writers’, such as methyltransferase-like 3/14/16 (METTL3/14/16) (11, 12), zinc finger CCCH-type containing 13 (ZC3H13) (13), vir-like m6A methyltransferase associated (VIRMA) (14), and RNA binding motif protein 15 (RBM15) (15), removed by m6A demethylases, aka ‘erasers’, such as fat mass and obesity-associated (FTO) (16) and alkB homolog 5 (ALKBH5) (17), and recognized by m6A reader proteins, aka ‘readers’, such as YTH domain family protein 1/2/3 (YTHDF1/2/3) (18–20), YTH domain containing 1/2 (YTHDC1/2) (21, 22). Recent studies have confirmed that liver function involving growth, development, metabolism and protein catabolic and development of liver diseases have been regulated by m6A RNA modification (23). For example, FTO, as an important m6A demethylases, is related to liver lipid metabolism and has been reported increase in the patients with liver of non-alcoholic fatty liver disease (NAFLD), leading to the reduced fatty acid oxidation and elevated lipid accumulation in human hepatocytes (24, 25). Additionally, dysregulation of m6A modification in HCC has also been reported (26). Ding et al. has demonstrated that increasing m6A modification of G-protein alpha-subunit (GNAS) mRNA in HCC cells *via* lipopolysaccharide (LPS) stimulation facilitates the expression of GNAS, contributing to the progression of the inflammation-related HCC (27). Therefore, it deserves our attention that the dynamic changes of m6A mRNA methylation in the process of HCC, especially in the early inflammation stage prior to tumor formation.

Interleukin-17A (IL-17A), one isoform of IL-17 family cytokines, is the prototypical member of this family, thus, researchers focused more on its proinflammatory role in autoimmune and inflammatory diseases (28). Previous studies showed that IL-17A is increased in a variety of liver diseases, including liver inflammatory diseases induced by hepatitis B/C (29, 30) and alcoholic liver disease (ALD) (31), and plays a role in intracellular signal transduction *via* binding to IL-17 receptor A (IL-17RA) (32), which is expressed on many cell types and stimulated by multiple IL-17 cytokines, including IL-17A, IL-17F, IL-17A/F, and IL-17E (IL-25) (33). In the liver, IL-17RA prefers to express on Kupffer cells (KCs), hepatic stellate cells (HSCs), biliary epithelial cells, sinusoidal endothelial cells and hepatocytes (34). Although IL-17RA-mediated inflammation in liver cells serves an important role in liver diseases, most previous studies focused on the IL-17RA signaling in non-parenchymal cells such as KCs and HSCs and indicated that IL-17RA-deficient KCs and HSCs showed impaired activation *in vivo* in response to the DEN-induced liver injury (35). Zhang et al. also found that down-regulation of IL-17RA suppressed the secretion of IL-6 induced by IL-17A in HSCs (36). However, recent studies indicated that mice devoid of IL-17RA in steatotic hepatocytes were protected from non-alcoholic steatohepatitis (NASH)- and ALD-induced HCC due to reduced inflammatory responses and suppression of cholesterol and fatty acid synthesis in steatotic hepatocytes (37), suggesting that IL-17RA signaling in steatotic hepatocytes is critical for the pathogenesis of NASH- and ALD-induced HCC and IL-17RA targeting therapies are likely to has the potential for the suppression of pathogenic inflammation.

Although current studies have noticed the significant role of IL-17RA-mediated inflammatory response in chronic hepatic inflammation, the exact molecular mechanisms underlying the increase of IL-17RA remains largely enigmatic. Therefore, given the pivotal role of m6A mRNA methylation in the initiation and development of HCC and the effect of IL-17RA signaling on the pathogenesis of NASH- and ALD-induced HCC, we performed methylated RNA immunoprecipitation sequencing (MeRIP-seq) using normal, HCC tumor and paired tumor adjacent tissues to investigate the dynamic changes of m6A mRNA methylation in the process of HCC and found the reduction of m6A mRNA methylation of IL-17RA in tumor adjacent tissues with chronic inflammation compared with normal liver tissues, suggesting the potential role IL-17RA in the inflammation-carcinogenesis transformation of HCC. Besides, we demonstrated that FTO functions as a main modulator for the decrease of m6A mRNA methylation of IL-17RA *via* knockdown and overexpression of FTO *in vitro* and *in vivo*.

## Materials and methods

### Clinical samples

All the frozen clinical samples (normal from the patients with hepatic hemangioma, HCC tumor and paired tumor adjacent tissues from the patients with hepatitis B virus infection) in this study were randomly collected from Eastern Hepatobiliary Surgery Hospital, Naval Medical University (Shanghai, China). Written informed consents were obtained from all patients amenable to the policies of the committee, and the study was approved by the Ethics Committee of Naval Medical University.

### Animal experiments

Wild-type C57BL/6 male mice were purchased from JiHui experimental animal Co. (Shanghai, China). For murine NAFLD model, the 5-week-old C57BL/6 male mice were assigned randomly to five groups (n = 5): control diet group (Control), high‐fat diet group (HFD, Dyets, 60% kcal from fat diet), HFD + 0.1% rhein diet group (HFD + Rhein, Dalian Meilun, China), HFD + AAV-NC group, and HFD + AAV-oeFTO group. The 5-week-old male mice were fed with these diets for a total of 14 weeks. For the murine chronic liver injury model, the 2-week-old male mice received intraperitoneal injection of 2 mg/kg N-nitrosodiethylamine (DEN) (CAS: 55-18-5, RHAWN). When the mice with DEN administration were 4 weeks old, mice received intraperitoneal injections of 20% CCl_4_ in olive oil at 5 ml/kg twice a week for 14 weeks and were fed with a control diet. For studies of FTO overexpression, each C57BL/6 male mouse was intravenously injected with a single dose of 1 × 10^11^ vector genomes of adeno-associated virus (AAV) serotype 8 carrying either FTO (AAV-oeFTO: pAAV-CMV-Fto-3FLAG) or control gene (AAV-NC: pAAV-CMV-MCS-3FLAG) *via* the portal vein. For better transfection, six weeks later after the injection, mice were started to use for further experiments. AAV-NC and AAV-oeFTO were constructed and purchased from OBiO Technology (Shanghai, China). All mice were housed under a 12 light/12dark cycle at a temperature of 21 ± 2 °C and with a humidity of 60 ± 5% at the animal facility of Naval Medical University. All experiments using mice were carried out following animal protocols approved by the Ethics Committee of Naval Medical University.

### Cell cultures

HCC-derived Huh-7 cell line was obtained from the Chinese Academy of Sciences Cell Bank (Shanghai, China) and cultured in Dulbecco’s modified Eagle’s medium (HyClone) containing 10% fetal bovine serum (Gibco, USA) and 1% penicillin/streptomycin at 37°C, in an atmosphere of 5% CO_2_.

### Generation of stable cell lines with FTO knockdown or overexpression

The lentiviral knockdown vector pLKD-CMV-G&PR-U6-shFTO (5′-GGAGCTCCATAAAGAGGTT-3′) and the lentiviral overexpression vector pLenti-CMV-FTO-3flag-PGK-EGFP-2A-Puro were constructed by OBiO Technology (Shanghai, China). Recombinant lentiviruses containing full-length FTO (oeFTO), FTO control (oeNC), shRNA targeting FTO (shFTO), and control shRNA (shNC) were used to transfect cells following the protocol provided by OBiO Technology (Shanghai, China). 2 mg/mL puromycin was used to select for stable cells.

### Plasmid construction and cell transfection

The wide type target site sequence and the 3′-UTR mutated sequence of the IL-17RA were synthesized and the plasmid overexpressing IL-17RA was constructed and provided by Genomeditech Co., LTD (Shanghai, China). IL-17RA was cloned into vector PGMLV-6751 (*Bam*HI, *Xho*I) (forward primer1, 5’-GCGAATTCGAAGTATACCTCGAGGCCACCATGGGGGCCGCAC-3’; reverse primer1, 5’-CCAGGCCCCGGAATTGGTTCTGGAGTGTCTGGCATTTCTG-3’; forward primer2, 5’-AATTCCGGGGCCTGGAAGTGAAAAATACAGTGATGAC-3’; reverse primer2, 5’-CGTCATGGTCTTTGTAGTCGGATCCTGCACTGGGCCCCTCTGACTC-3’). The wild type plasmid overexpressing IL-17RA (PGMLV-CMV-H_IL17RA-3×Flag-EF1-ZsGreen1-T2A-Puro) was named oe_IL-17RA_WT, and site-directed mutants plasmid (PGMLV-CMV-H_IL17RA (c.A2319C)-3×Flag-EF1-ZsGreen1-T2A-Puro) overexpressing IL-17RA was named oe_IL-17RA_MUT. (The cloned sequences are listed in **Supplementary Tables S1, 2**). The empty plasmids were transfected as negative controls. For cell transfection, Huh7 cells were cultured in 6-well plates to reach a confluence of 70 – 90% and then transfected with IL-17RA overexpression plasmids (WT and MUT) and NC plasmids using Lipofectamine 3000 reagent (Invitrogen) according to the manufacturer’s instructions. After 48 hours of transfection, transfection efficiency was detected by quantitative real-time polymerase chain reaction (qRT-PCR) and Western blot.

### MeRIP-seq

MeRIP-seq was performed as described previously by Shanghai Jiayin Biotechnology Ltd (Shanghai, China) (38). In brief, total RNA was isolated and fragmented into ~100-nucleotide-long fragments. Approximately 5% of fragmented RNA as input RNA, other RNA was analyzed by immunoprecipitation using affinity-purified anti-m6A polyclonal antibodies (ABE572, Millipore, Germany). Sequencing was carried out using an Illumina NovaSeq 6000 platform. The method of analysis is based on the previously described m6A-seq protocol.

### RNA immunoprecipitation

The RIP experiment was conducted with the Magna RIP RNA-Binding Protein Immunoprecipitation Kit (Millipore, USA) following the manufacturer’s instructions. The m6A antibody (synaptic systems) and anti-rabbit IgG (Millipore, USA) were used for the RIP experiment. Total RNA was performed as input control. The interested RNAs were detected by qPCR. Relative enrichment was normalized to the input: %Input =1/10 × 2^Ct [IP] – Ct [input]^.

### Western-blot analysis

Total protein was extracted from the frozen clinical tissues, murine model tissues or cells with radioimmunoprecipitation assay (RIPA) Lysis Buffer and PMSF (Beyotime, Shanghai, China). The protein was electrophoresed on 7.5%-12% SDS–polyacrylamide gels according to the different proteins molecular weight and transferred to nitrocellulose membranes (Bio-Rad, Hercules, USA). The membranes were incubated with the anti-IL-17RA receptor antibody, anti-FTO antibody, anti-ALKBH5 antibody (Abcam, Cambridge, USA), anti-METTL3 antibody (Zen bio, China), anti-METTL14 antibody (Sigma-Aldrich, Germany), β-Actin antibody (Proteintech, USA) overnight at 4°C. And then the membranes were incubated with secondary antibody IRdye 680RD goat (polyclonal) anti-mouse IgG (H+L) and IRdye 800CW goat (polyclonal) anti-rabbit IgG (H+L) (Licor Biosciences, Nebraska, USA) for 2 hours at room temperature. The membranes were detected using an Odyssey infrared scanner (Licor Biosciences, Nebraska, USA). The antibodies are listed in **Table S3**.

### RNA extraction and qRT-PCR analysis

Total RNA from the frozen clinical tissues, murine models, or cells were extracted with Trizol reagent (Takara, Dalian, China). For reverse transcriptions, cDNA was generated using a Reverse Transcription Kit (Takara, Dalian, China). Quantitative real-time PCR was performed using SYBR^®^ Green (Takara, Dalian, China) in a StepOne™ Real-Time PCR System (Applied Biosystems). β-actin was employed as an endogenous control. The relative mRNA levels were calculated using the comparative Ct method. The PCR primers are listed in **Table S4**.

### Histological staining

Liver tissues from murine models were fixed with 4% paraformaldehyde (Servicebio, China) for more than 24 hours, then embedded in paraffin and sectioned at 4-5 μm slices. For H&E staining, hematoxylin and eosin were used in the micro-section to analyze the histopathological damage in the liver from all experiment groups. For Sirius red staining, the slices were stained with picrosirius red solution for 8-10 min and then dehydrated and mounted in xylene. For Oil red O staining, lipid droplet accumulation was stained using 8-10 μm of frozen liver slices following a standard protocol. All the images were reviewed under the microscope (Eclipse Ci-L, Nikon, Japan) at 20× magnification and analyzed under Image-Pro Plus 6.0 (Media Cybemetics, USA) by Servicebio company (Wuhan, China). The NAFLD activity score (NAS) was calculated from the sum of the individual scores for steatosis, inflammation and ballooning. The NAFLD activity score is listed in **Table S5**.

### Determination of serum/tissue biochemical indicators

The obtained mice blood was centrifuged at 3000 × *g* for 10 min, and the supernatant was collected. The serum levels of alanine aminotransferase (ALT), aspartate aminotransferase (AST), triglyceride (TG), and the tissues levels of TG and hydroxyproline (HYP) were measured by using ALT assay kit (C009-2), AST assay kit (C010-2), TG assay kit (A110-1), and HYP assay kit (A030-2) following Nanjing Jiancheng Institute of Biotechnology protocols (Jiangsu, China).

### Multiplex cytokine assay

Mouse serum was prepared by centrifugation at 1000 × g for 10 min at 4°C and stored in aliquots at −80°C until analysis. Cytokine concentration was measured in all of the mouse serum samples using a Mouse High Sensitivity T Cell Magnetic Bead Panel (MHSTCMAG-70K-08, EMD Millipore, Billerica, MA, USA) on the Luminex 100/200 multiplex immunoassay system according to the manufacturer’s instructions. The data were analyzed using Belysa 1.0 software (luminex 200).

### Statistical analyses

All experiments were repeated at least three times and expressed as the means ± SEM. SPSS (version 17.0) software was used to analyze the data in this study. For comparisons involving three groups, data were analyzed using a one-way analysis of variance (ANOVA) followed by the least significant difference (LSD) test. For comparisons involving only two groups, data were analyzed with Student’s t test (two-tailed). R software (version 4.0.3) including ggplot2 and pheatmap packages was used for plot drawing. The following data significance levels were used for comparisons between independent groups: ns, not significant; *, *p* < 0.05; **, *p* < 0.01; ***, *p* < 0.001; ****, *p* < 0.0001.

## Results

### MeRIP-seq reveals the reduction of m6A mRNA methylation of IL-17RA in tumor adjacent tissues

**Figure 1:**
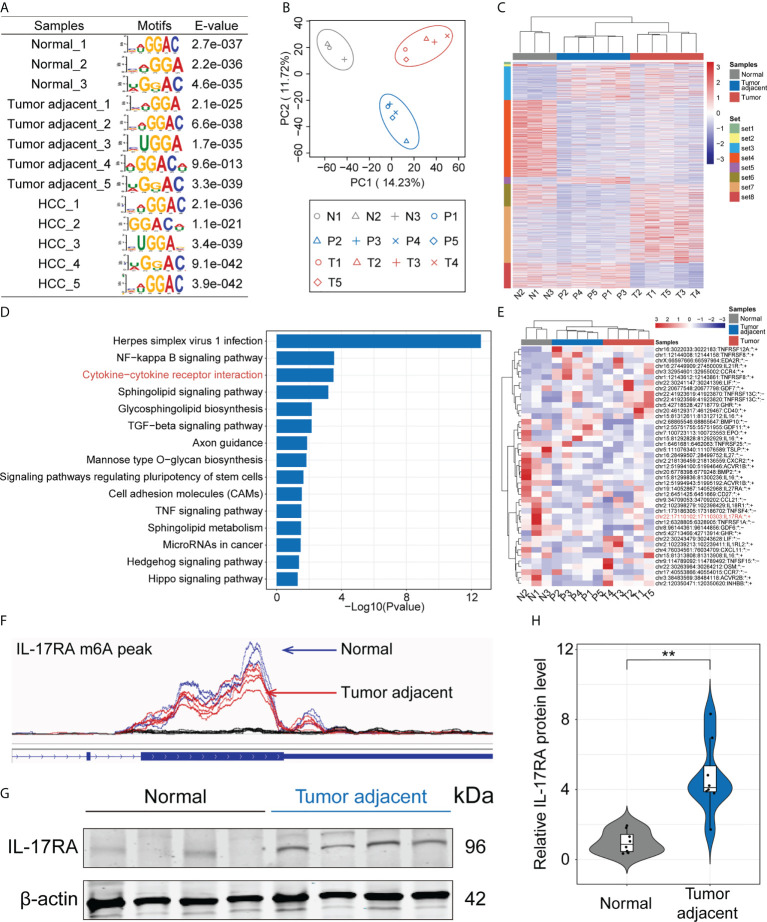
Dissecting the role of m6A mRNA methylation in the progression of HCC by MeRIP-seq. **(A)** Enriched RNA motifs from the m6A MeRIP-seq data within each sample. **(B)** PCA analysis of all samples. **(C)** Heatmap of m6A methylation levels of all samples. **(D)** KEGG analysis of differentially m6A modified genes. **(E)** Heatmap of differentially m6A modified genes related to the cytokine-cytokine receptor interaction. **(F)** m6A mRNA methylation level of IL-17RA between normal and tumor-adjacent liver tissues. **(G)** Western blot for the expression of IL-17RA in normal liver and tumor-adjacent liver tissues of patients. **(H)** Quantifications of IL-17RA expression in different tissues by Western blot. **p < 0.01.

To investigate the dynamic changes and potential mechanisms of m6A modification during the progression of HCC, we performed MeRIP-seq using normal, HCC tumor and paired tumor adjacent tissues. The m6A consensus modified motif GGAC was identified in all samples obtained based on E-value (**Figure 1A**). Then, we evaluated dimensionality reduction on the sequencing data by principal component analysis (PCA), and results showed that three kinds of samples could be classified accurately (**Figure 1B**). Heatmap of m6A methylation levels also showed significant differences among the three kinds of samples (**Figure 1C**). To further explore the function of differentially m6A modified genes in the progression of HCC, KEGG (Kyoto Encyclopedia of Genes and Genomes) analysis was performed. The top three pathways including herpes simplex virus 1 infection, NF-kappa B signaling pathway and cytokine-cytokine receptor interaction were significantly enriched (**Figure 1D**). Considering the important role of cytokines during the process of HCC (39), we took a closer look at the genes of m6A modification related to the cytokine-cytokine receptor interaction by heatmap and found that these genes were significantly decreased in tumor adjacent and HCC tumor tissues (**Figure 1E**). Compared with normal liver tissues, there were no significant difference between tumor adjacent tissues and HCC tumor tissues, especially IL-17RA (**Figure 1E**). Mapping of m6A methylation also revealed that m6A mRNA methylation level of IL-17RA exhibited a significant decrease in the tumor adjacent tissues, compared with normal tissues (**Figure 1F**). Importantly, the upregulation of IL-17RA in the tumor adjacent tissues was confirmed at protein level determined by Western blot (**Figures 1G, H**). Together, these results suggested that m6A mRNA methylation might be responsible for the abnormal expression of cytokine receptor IL-17RA in human livers, contributing to liver inflammation and fibrosis.

### FTO functions as a main modulator for the decrease of m6A mRNA methylation of IL-17RA

**Figure 2:**
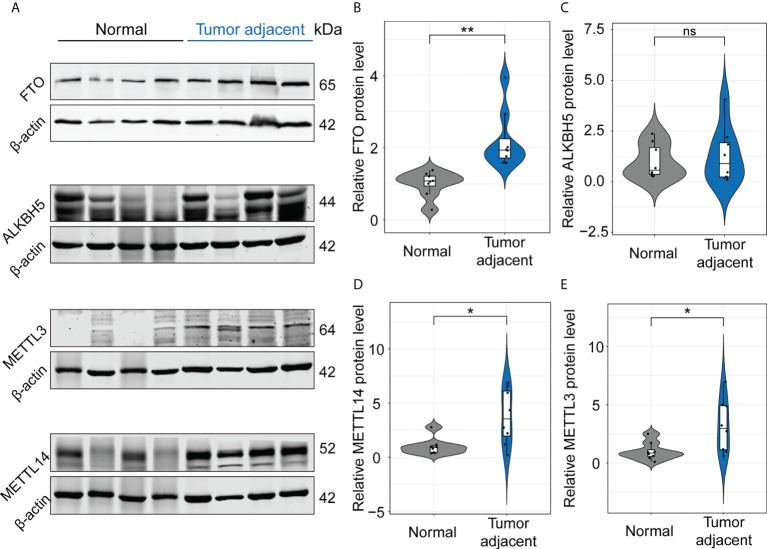
FTO functions as a main modulator for the decrease of m6A mRNA methylation of IL-17RA. **(A)** Western blot for the expression of FTO, ALKBH5, METTL3, and METTL14 in normal liver and tumor-adjacent liver tissues of patients. **(B-E)** Quantifications of FTO, ALKBH5, METTL14, and METTL3 expression in different tissues by Western blot. All experiments were repeated at least three times and expressed as the means ± SEM. ns, not significant; *p < 0.05; **p < 0.01.

The above MeRIP-seq data suggested that m6A mRNA methylation of IL-17RA was significantly decreased in the tumor adjacent tissues. In order to unravel the driving forces behind the markedly changed m6A patterns in the tumor adjacent tissues, we analyzed the expression of proteins related to coordinate m6A modifications by Western blot, including m6A methylation writers (METTL3, METT14) and erasers (FTO, ALKBH5) (**Figure 2A**). Compared with normal tissues, the upregulation of FTO in the tumor adjacent tissues was confirmed at protein level (**Figure 2B**), while the expression of ALKBH5 showed no significant difference between normal and tumor adjacent tissues (**Figure 2C**). Besides, the expression of METTL14 and METTL3 at protein level were significantly upregulated in the tumor adjacent tissues (**Figures 2D, E**). Therefore, these results showed that FTO is a main reason for the decrease of m6A mRNA methylation of IL-17RA.

### Regulation of FTO interferes protein level of IL-17RA *in vitro*

**Figure 3:**
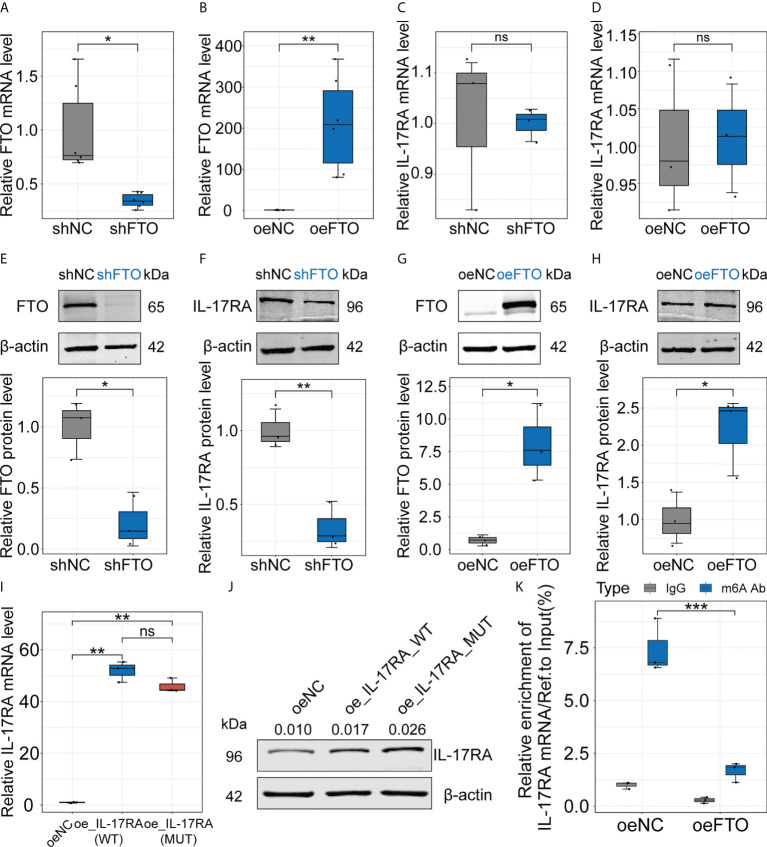
Regulation of FTO interferes protein level of IL-17RA *in vitro*. **(A-D)**. Relative FTO or IL-17RA mRNA level in Huh7 cells after FTO knockdown or overexpression. **(E-H)**. Relative FTO or IL-17RA protein level in Huh7 cells after FTO knockdown or overexpression. **(I)**. Relative IL-17RA mRNA level in Huh7 cells after IL-17RA_WT or IL-17RA_MUT overexpression. **(J)**. IL-17RA protein level in Huh7 cells after IL-17RA_WT or IL-17RA_MUT overexpression. **(K)**. Relative m6A enrichment of IL-17RA mRNA in Huh7 cells after FTO overexpression by m6A-RIP-qPCR. All experiments were repeated at least three times and expressed as the means ± SEM. ns, not significant; *p < 0.05; **p < 0.01; ***p < 0.001.

To further investigate the relationship between FTO and IL-17RA, we utilized Huh7 cells to construct cell lines stably underexpressing or overexpressing FTO using lentiviral vectors and the results showed the decreased and markedly increased FTO expression at transcript and protein levels, respectively (**Figures 3A, B, E, G**). Then, the mRNA level of IL-17RA was examined, which indicated no significant change in shFTO or oeFTO cell lines (**Figures 3C, D**). However, the protein level of IL-17RA extremely decreased in shFTO cell line and elevated in oeFTO cell line, implying the existence of post-transcriptional modification of IL-17RA (**Figures 3F, H**). Therefore, the wild type plasmid overexpressing IL-17RA (oe_IL-17RA_WT) and the site-directed mutants (in the site of m6A modification of IL-17RA) plasmid overexpressing IL-17RA (oe_IL-17RA_MUT) were constructed and transinfected into Huh7 cells, the result of which showed no obvious change at transcript level between cells with oe_IL-17RA_WT and oe_IL-17RA_MUT (**Figure 3I**). On the contrary, cells with oe_IL-17RA_MUT exhibited higher protein expression of IL-17RA compared with cells of oe_IL-17RA_WT (**Figure 3J**). Meanwhile, we found relative m6A enrichment of IL-17RA mRNA reduced in Huh7 cells after FTO overexpression by m6A-RIP-qPCR (**Figure 3K**). Taken together, the results above demonstrated the protein of IL-17RA was regulated by FTO *via* m6A modification *in vitro*.

### FTO promotes liver damage in murine NAFLD model

**Figure 4:**
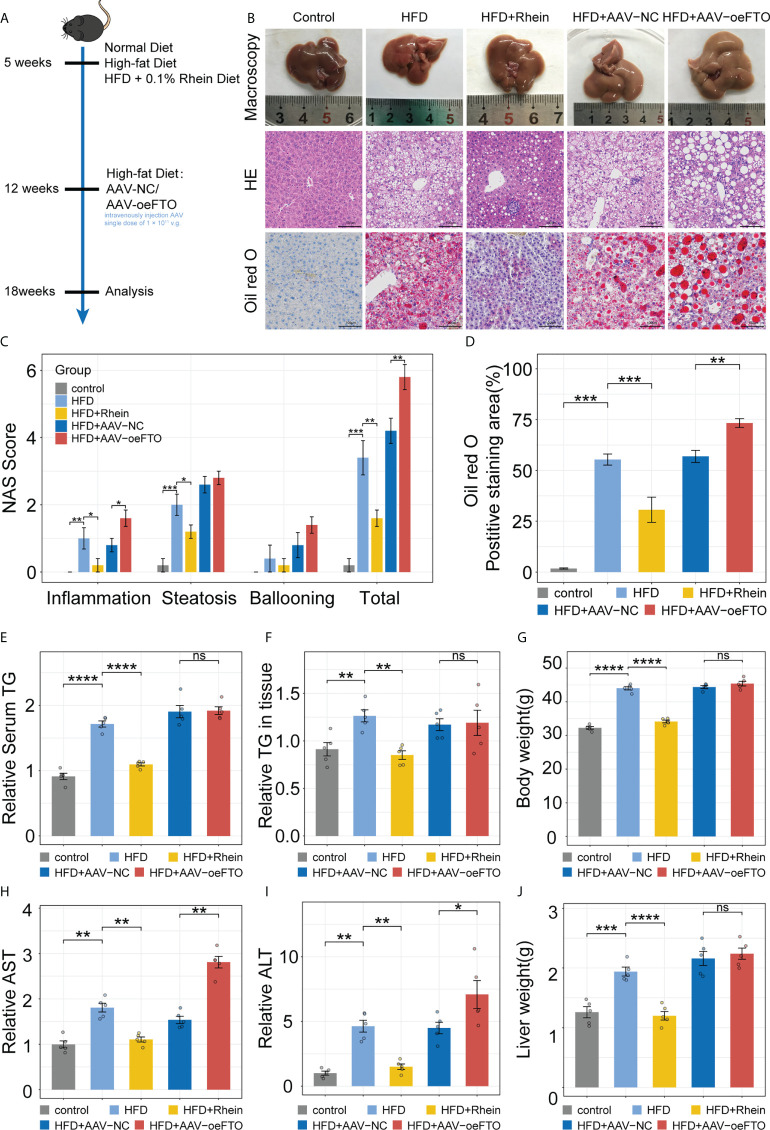
FTO promotes liver damage in murine NAFLD model. **(A)** Schematic of the *in vivo* experimental setup for construction of murine NAFLD model to ascertain FTO function. **(B)** Representative images of liver tissues in groups with different treatment by macroscopy and light microscopy (H&E staining and Oil red O staining). **(C)** NAFLD Activity Score (NAS), including inflammation, steatosis, ballooning, and total NAS in all groups with different treatment. **(D)** Quantitative analysis of Oil red O intensity in all groups with different treatment. **(E)** Relative serum TG in all groups with different treatment. **(F)** Relative TG level in liver tissues of all groups with different treatment. **(G)** Body weight of mice in all groups with different treatment. **(H, I)**. Relative serum AST, and ALT in all groups with different treatment. **(J)** Liver weight of mice in all groups with different treatment. All experiments were repeated at least three times and expressed as the means ± SEM. n = 5; ns, not significant; *p < 0.05; **p < 0.01; ***p < 0.001; ****p < 0.0001.

In order to further verify that the modulator function of FTO on IL-17RA *in vivo*, murine NAFLD model was established as performed previously (**Figure 4A**). Compared with HFD group, NAS score of H&E staining in HFD + Rhein [inhibitor of FTO (40)] group showed apparently lower, manifesting in reduced inflammation infiltration and steatosis (**Figures 4B, C**). Meanwhile, positive staining area of Oil red O used for estimation of liver fat accumulation in mice group with HFD treated was at almost twice the group with HFD + Rhein (**Figure 4D**). Indeed, treatment with Rhein not only reduced TG level in serum and liver tissues, but the level of ALT and AST level as well (**Figures 4E, F, H, I**). Of note, Rhein feeding unexpectedly reduced the body weight and liver weight of the mice, which exhibited no significant change compared with the control group, suggesting the probably functional role of Rhein in the people of HFD (**Figures 4G, J**). Contrarily, mice administrated with AAV-oeFTO *via* intravenous injection performed higher total NAS score, positive staining area of Oil red O, ALT and AST level compared with HFD + AAV-NC group (**Figures 4C, D, H, I**). In addition, mice overexpressing FTO showed no significant change in TG, body weight and liver weight in comparison to HFD + AAV-NC group (**Figures 4E, F, G, J**). Consequently, we confirmed the functional role of FTO in liver damage in murine NAFLD model.

### FTO facilitates liver inflammation *via* induction of IL-17RA in murine NAFLD model

**Figure 5:**
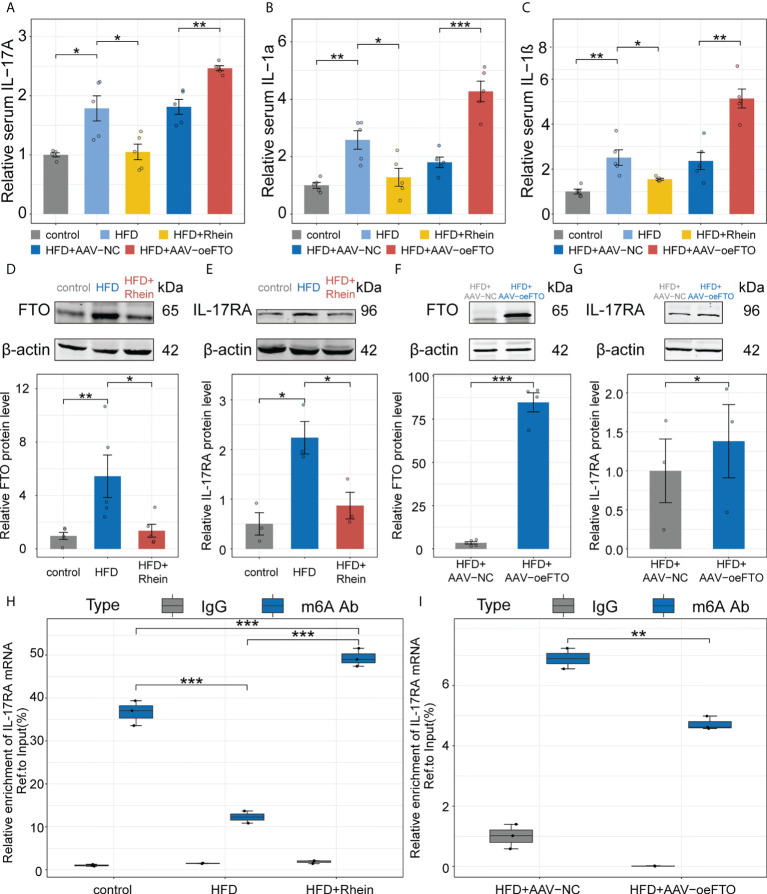
FTO facilitates liver inflammation *via* induction of IL-17RA in murine NAFLD model. **(A-C)**. Relative serum IL-17A, IL-1α, and IL-1β in all groups with different treatment. **(D, E)**. Relative FTO, and IL-17RA protein level in the control, HFD, and HFD + Rhein groups. **(F, G)**. Relative FTO, and IL-17RA protein level in the HFD + AAV-NC, and HFD + AAV-oeFTO groups. **(H, I)**. Relative m6A enrichment of IL-17RA mRNA in liver tissues of different groups by m6A-RIP-qPCR. All experiments were repeated at least three times and expressed as the means ± SEM. n = 5; *p < 0.05; **p < 0.01; ***p < 0.001.

Besides the markers related to liver damage, we also examined certain inflammatory factors including IL-17A, IL-1α and IL-1β in murine NAFLD model and results showed treatment with Rhein obviously suppressed the increase of IL-17A, IL-1α and IL-1β due to the high-fat diet in murine NAFLD model (**Figures 5A–C**). Reversely, mice overexpressing FTO using AAV-oeFTO vector exhibited higher levels of proinflammatory factors (**Figures 5A–C**). Additionally, the relationship between FTO and IL-17RA was determined as well *in vivo*. Consistent with the results *in vitro*, FTO protein level was suppressed in HFD + Rhein treatment group and in the meantime, the expression of IL-17RA also decreased (**Figures 5D, E**). On the contrary, overexpression of FTO contributed to the increase of IL-17RA (**Figures 5F, G**). Then, m6A-RIP-qPCR was performed to quantify the m6A methylation modification of IL-17RA and we found that the reduction of FTO by Rhein led to the higher relative m6A enrichment of IL-17RA mRNA and vice versa (**Figures 5H, I**). Thus, we concluded that FTO facilitates the expression of IL-17RA *via* suppression of m6A methylation modification to accelerate the liver inflammation in murine NAFLD model.

### FTO facilitates liver injury and inflammation *via* induction of IL-17RA in murine chronic liver injury model

**Figure 6:**
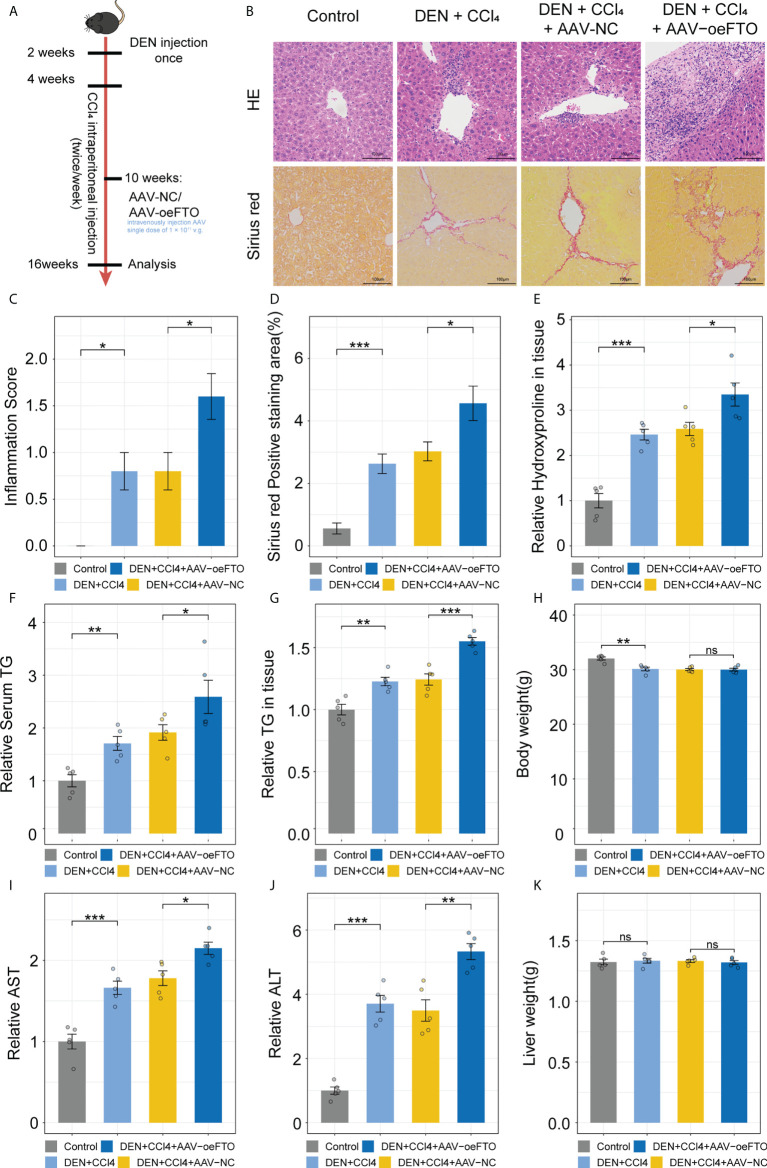
FTO promotes liver damage in murine chronic liver injury model. **(A)** Schematic of the *in vivo* experimental setup for construction of murine chronic liver injury model to ascertain FTO function. **(B)** Representative images of liver tissues staining for H&E and Sirius red in groups with different treatment by light microscopy. **(C)** Inflammation score in all groups with different treatment. **(D)** Quantitative analysis of Sirius red positive area in all groups with different treatment. **(E)** Relative hydroxyproline level in liver tissues of all groups with different treatment. **(F)** Relative serum TG in all groups with different treatment. **(G)** Relative TG level in liver tissues of all groups with different treatment. **(H)** Body weight of mice in all groups with different treatment. **(I, J)**. Relative serum AST, and ALT in all groups with different treatment. **(K)** Liver weight of mice in all groups with different treatment. All experiments were repeated at least three times and expressed as the means ± SEM. n = 5; ns, not significant; *p < 0.05; **p < 0.01; ***p < 0.001.

**Figure 7:**
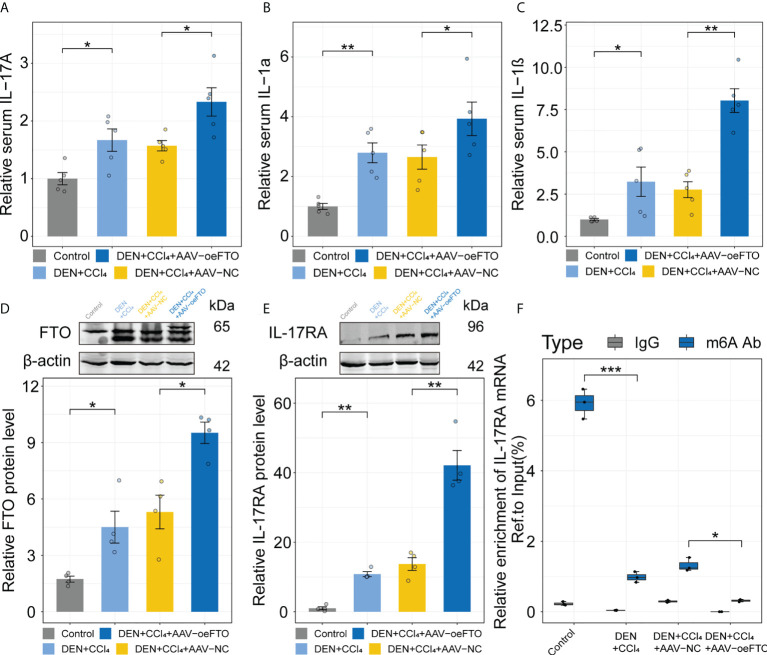
FTO facilitates liver inflammation *via* induction of IL-17RA in murine chronic liver injury model. **(A-C)**. Relative serum IL-17A, IL-1α, and IL-1β in all groups with different treatment. **(D, E)**. Relative FTO, and IL-17RA protein level in the control, DEN + CCl_4_, DEN + CCl_4_ + AAV-NC, and DEN + CCl_4_ + AAV-oeFTO groups. **(F)**. Relative m6A enrichment of IL-17RA mRNA in liver tissues of different groups by m6A-RIP-qPCR. All experiments were repeated at least three times and expressed as the means ± SEM. n = 5; *p < 0.05; **p < 0.01; ***p < 0.001.

Similar results showing FTO contributes to liver damage were obtained using another model of DEN + CCl_4_-induced chronic liver injury in murine (**Figure 6A**). Inflammation score of H&E staining in the group exposure to DEN + CCl_4_ increased significantly compared with the control group and addition of AAV containing full-length FTO further aggravated the condition (**Figures 6B, C**). Besides, positive staining area of Sirius red used for estimation of liver fibrosis in mice group with DEN + CCl_4_ + AAV-oeFTO treated was bigger than the counterpart (**Figure 6D**). In fact, overexpression of FTO by AAV also promoted the level of hydroxyproline, TG, ALT and AST in liver tissues or serum (**Figures 6E–G, I, J**), but had no effect on the body and liver weight of mice (**Figures 6H, K**). In line with the murine NAFLD model, mice in DEN + CCl_4_ + AAV-oeFTO group displayed higher IL-17A, IL-1α and IL-1β level in serum (**Figures 7A–C**). Similarly, we found a positive correlation relation between the expression of FTO and IL-17RA (**Figures 7D, E**). In addition, the result of m6A-RIP-qPCR showed a significant reduction of m6A methylation modification of IL-17RA in DEN + CCl_4_ + AAV-oeFTO group, suggesting that FTO could force the translation of IL-17RA *via* downregulation of methylation level of IL-17RA mRNA (**Figure 7F**).

## Discussion

Several MeRIP-seq studies have extended our knowledge of molecular pathogenesis of HCC (41, 42). However, little is known about the association of m6A modification and hepatic inflammation, which is recognized as the most important stage in the inflammation-carcinogenesis transformation of HCC. To our knowledge, this study firstly identified the positive relationship between FTO and IL-17RA in the chronic hepatic inflammation. FTO overexpression increased the demethylation of IL-17RA mRNA, thus resulting in the elevated protein level of IL-17RA. By contrast, FTO suppression inhibited the expression of IL-17RA and alleviated the hepatic inflammation in murine models, which provided a promising target for the therapeutic regimen in the reversal of inflammation-carcinogenesis transformation of HCC.

Recent studies have revealed the presence of differential m6A RNA methylation in mice fatty liver, and showed that hypermethylated genes were enriched in the processes associated with hepatic lipid metabolism and hypomethylated genes were related to translation-associated processes (43). In this study, MeRIP-seq was performed using normal, HCC tumor and paired tumor adjacent tissues to investigate the dynamic changes of m6A mRNA methylation in the inflammation-carcinogenesis transformation of HCC and we found the change of m6A mRNA modification of IL-17RA between normal liver tissues and tumor adjacent tissues. However, m6A mRNA modification of IL-17RA between tumor adjacent tissues and HCC tumor tissues showed little difference, indicating that the m6A mRNA demethylation of IL-17RA has become a critical event in the early stage of HCC prior to tumor formation.

Chronic hepatic inflammation induced by either hepatitis virus or metabolic diseases has been recognized as a key factor progressing from hepatitis to hepatocarcinogenesis. Previous studies have described the pivotal role of IL-17 signaling axis in many liver diseases, such as ALD, chronic hepatitis B/C, and NFALD. Notably, Tang et al. has demonstrated that IL-17A induced hepatocyte steatosis and promoted the IL-6 production by hepatocyte (44). While IL-17RA, a receptor for IL-17A, is extensively expressed on many cells and its function has been best outlined in IL-17 family receptor, its expression on hepatocytes has not been well characterized (45). In our study, compared with the normal liver tissues, we found the increased expression of IL-17RA in tumor adjacent tissues. Additionally, the elevated expression of IL-17RA was also validated through *in vivo* assays of murine NAFLD model and murine chronic liver injury model.

Given that the declining m6A mRNA methylation and increasing translational level of IL-17RA in tumor adjacent tissues, four critical regulators associated with m6A modification including FTO, ALKBH5, METTL14, and METTL3 were examined and results showed FTO, the m6A methyltransferase, was considered as the most important modulator in our study by Western blot. Consistent with the study of Lim et al (46), which has demonstrated that elevated FTO exists in the livers of NASH patients and rodent NASH model and suppression of FTO alleviates palmitic acid-induced lipotoxicity, we also found both NASH induced by HFD and chronic hepatic inflammation induced by chemicals upregulated the expression of FTO. In terms of mechanisms, a series of assays *in vitro* and *in vivo* were designed to determine the positive relationship between FTO and IL-17RA. Regulation of FTO in Huh7 cells by lentivirus obviously interferes the translational level of IL-17RA. In the meantime, the site-directed mutants (in the site of m6A modification of IL-17RA) plasmid overexpressing IL-17RA (oe_IL-17RA_MUT) was constructed and transinfected into Huh7 cells and the result showed there was no difference at the level of mRNA compared with the wild type plasmid, whereas the protein expression of IL-17RA was elevated in Huh7 cells expressing oe_IL-17RA_MUT, indicating that m6A mRNA demethylation of IL-17RA promoted its protein translation. Furthermore, in both murine chronic hepatitis models induced by HFD or chemicals, we demonstrated that overexpression of FTO facilitates the liver injury and inflammation *via* induction of IL-17RA. Of note, overexpression of FTO in both murine models also gave rise to the elevated expression of cytokine IL-17A, which could bind to the increasing IL-17RA and lead to a more serious inflammation and liver injury.

Nevertheless, our study had some limitations and several improvements also need to be acquired due to the insufficient points in our study. The decreased m6A mRNA modification of IL-17RA in tumor adjacent tissues of the liver may require a larger cohort to validate. Additionally, relative IL-17RA mRNA level has no significant change between the cells of FTO knockdown or overexpression and the counterpart, however, the IL-17RA protein level in two cell lines exhibited opposite. Therefore, further studies are needed to explore the mechanism that the decreased m6A mRNA methylation of the IL-17RA resulted in the increased protein level of IL-17RA in chronic hepatic inflammation.

## Conclusion

In this study, we demonstrated that m6A mRNA modification functions as an important modulator in chronic hepatic inflammation. FTO elevated in tumor adjacent tissues of the liver and contributed to the m6A mRNA demethylation of IL-17RA, which led to the elevated translational regulation of IL-17RA, exacerbating liver inflammation and worsening liver function. Further research showed FTO suppression alleviated the hepatic inflammation response through *in vivo* assays. These findings not only extend our insights into the molecular mechanisms underlying HCC pathogenesis, but also provide a promising therapy to improve chronic hepatic inflammation and prevent the initiation of HCC.

## Data availability statement

The original contributions presented in the study are included in the article/**Supplementary Materials**. Further inquiries can be directed to the corresponding authors.

## Ethics statement

The studies involving human participants were reviewed and approved by ethics committee of Naval Medical University. The patients/participants provided their written informed consent to participate in this study. The animal study was reviewed and approved by Ethics Committee of Naval Medical University. Written informed consent was obtained from the individual(s) for the publication of any potentially identifiable images or data included in this article.

## Author contributions

XG contributed to writing the original draft and data curation; ZD and CG contributed to formal analysis, data curation, and methodology; HY, YW, and JT contributed to performing the experiments; SS, WZ, SY, and FY contributed to supervision; FY and XG contributed to conceptualization, writing (original draft, review, and editing), formal analysis, and data curation. All authors contributed to the article and approved the submitted version.

## Funding

This research was supported by grants from the National Natural Science Foundation of China (nos. 81972657, 81830085 and 81972575); National Key Research and Development Program of China (2016YFC1302303); Shanghai Rising-Star Program (19QA1408700).

## Acknowledgments

Thanks to the members of our laboratory for their contributions.

## Conflict of interest

The authors declare that the research was conducted in the absence of any commercial or financial relationships that could be construed as a potential conflict of interest.

## Publisher’s note

All claims expressed in this article are solely those of the authors and do not necessarily represent those of their affiliated organizations, or those of the publisher, the editors and the reviewers. Any product that may be evaluated in this article, or claim that may be made by its manufacturer, is not guaranteed or endorsed by the publisher.
