# Supplementary material for: FTO promotes liver inflammation by suppressing m6A mRNA methylation of IL-17RA
Source: Front Oncol. 2022 Sep 12;12:989353. doi: 10.3389/fonc.2022.989353 (PMC9511030; doi:10.3389/fonc.2022.989353)
Supplement: Supplementary file 1 [file DataSheet_1.docx]

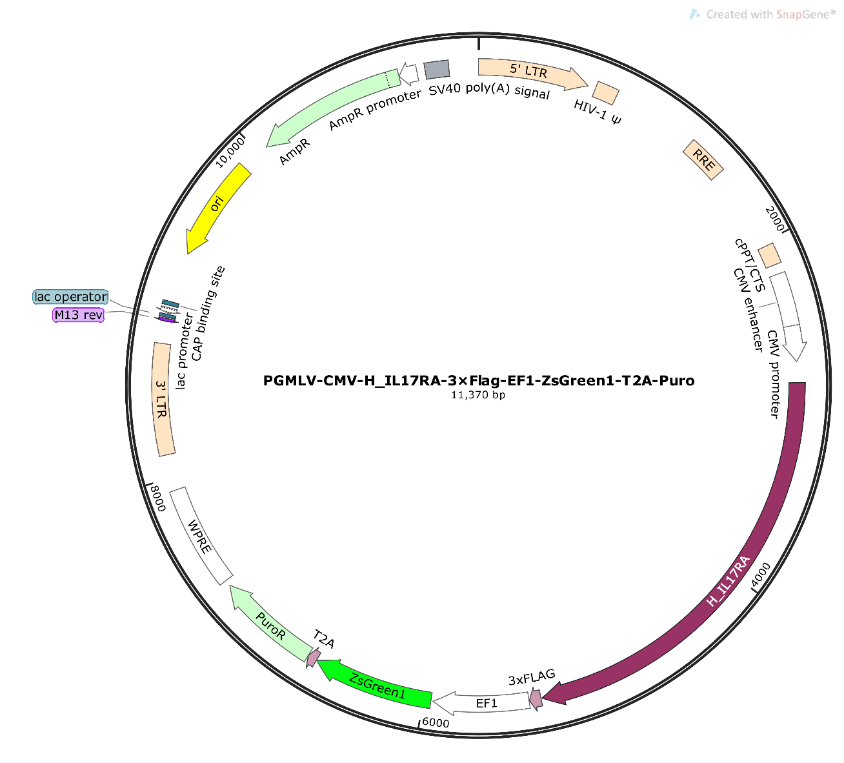

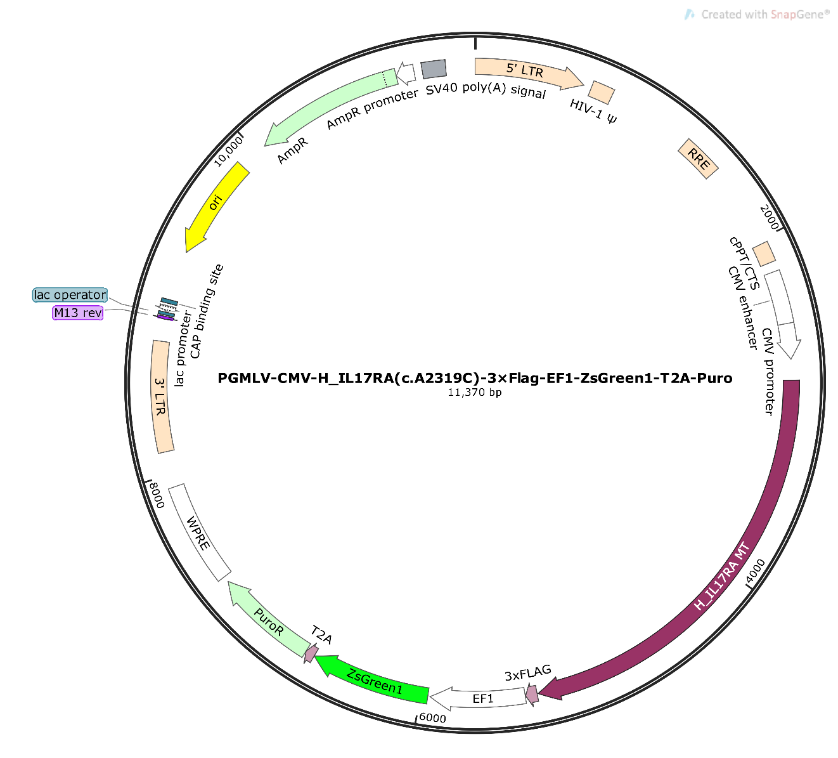


Table S1. The list of wide type sequence of the IL17RA.

| plasmid | PGMLV-CMV-H_IL17RA-3×Flag-EF1-ZsGreen1-T2A-Puro |
| --- | --- |
| GCCACCATGGGGGCCGCACGCAGCCCGCCGTCCGCTGTCCCGGGGCCCCTGCTGGGGCTGCTCCTGCTGCTCCTGGGCGTGCTGGCCCCGGGTGGCGCCTCCCTGCGACTCCTGGACCACCGGGCGCTGGTCTGCTCCCAGCCGGGGCTAAACTGCACGGTCAAGAATAGTACCTGCCTGGATGACAGCTGGATTCACCCTCGAAACCTGACCCCCTCCTCCCCAAAGGACCTGCAGATCCAGCTGCACTTTGCCCACACCCAACAAGGAGACCTGTTCCCCGTGGCTCACATCGAATGGACACTGCAGACAGACGCCAGCATCCTGTACCTCGAGGGTGCAGAGTTATCTGTCCTGCAGCTGAACACCAATGAACGTTTGTGCGTCAGGTTTGAGTTTCTGTCCAAACTGAGGCATCACCACAGGCGGTGGCGTTTTACCTTCAGCCACTTTGTGGTTGACCCTGACCAGGAATATGAGGTGACCGTTCACCACCTGCCCAAGCCCATCCCTGATGGGGACCCAAACCACCAGTCCAAGAATTTCCTTGTGCCTGACTGTGAGCACGCCAGGATGAAGGTAACCACGCCATGCATGAGCTCAGGCAGCCTGTGGGACCCCAACATCACCGTGGAGACCCTGGAGGCCCACCAGCTGCGTGTGAGCTTCACCCTGTGGAACGAATCTACCCATTACCAGATCCTGCTGACCAGTTTTCCGCACATGGAGAACCACAGTTGCTTTGAGCACATGCACCACATACCTGCGCCCAGACCAGAAGAGTTCCACCAGCGATCCAACGTCACACTCACTCTACGCAACCTTAAAGGGTGCTGTCGCCACCAAGTGCAGATCCAGCCCTTCTTCAGCAGCTGCCTCAATGACTGCCTCAGACACTCCGCGACTGTTTCCTGCCCAGAAATGCCAGACACTCCAGAACCAATTCCGGGGCCTGGAAGTGAAAAATACAGTGATGACACCAAATACACCGATGGCCTGCCTGCGGCTGACCTGATCCCCCCACCGCTGAAGCCCAGGAAGGTCTGGATCATCTACTCAGCCGACCACCCCCTCTACGTGGACGTGGTCCTGAAATTCGCCCAGTTCCTGCTCACCGCCTGCGGCACGGAAGTGGCCCTGGACCTGCTGGAAGAGCAGGCCATCTCGGAGGCAGGAGTCATGACCTGGGTGGGCCGTCAGAAGCAGGAGATGGTGGAGAGCAACTCTAAGATCATCGTCCTGTGCTCCCGCGGCACGCGCGCCAAGTGGCAGGCGCTCCTGGGCCGGGGGGCGCCTGTGCGGCTGCGCTGCGACCACGGAAAGCCCGTGGGGGACCTGTTCACTGCAGCCATGAACATGATCCTCCCGGACTTCAAGAGGCCAGCCTGCTTCGGCACCTACGTAGTCTGCTACTTCAGCGAGGTCAGCTGTGACGGCGACGTCCCCGACCTGTTCGGCGCGGCGCCGCGGTACCCGCTCATGGACAGGTTCGAGGAGGTGTACTTCCGCATCCAGGACCTGGAGATGTTCCAGCCGGGCCGCATGCACCGCGTAGGGGAGCTGTCGGGGGACAACTACCTGCGGAGCCCGGGCGGCAGGCAGCTCCGCGCCGCCCTGGACAGGTTCCGGGACTGGCAGGTCCGCTGTCCCGACTGGTTCGAATGTGAGAACCTCTACTCAGCAGATGACCAGGATGCCCCGTCCCTGGACGAAGAGGTGTTTGAGGAGCCACTGCTGCCTCCGGGAACCGGCATCGTGAAGCGGGCGCCCCTGGTGCGCGAGCCTGGCTCCCAGGCCTGCCTGGCCATAGACCCGCTGGTCGGGGAGGAAGGAGGAGCAGCAGTGGCAAAGCTGGAACCTCACCTGCAGCCCCGGGGTCAGCCAGCGCCGCAGCCCCTCCACACCCTGGTGCTCGCCGCAGAGGAGGGGGCCCTGGTGGCCGCGGTGGAGCCTGGGCCCCTGGCTGACGGTGCCGCAGTCCGGCTGGCACTGGCGGGGGAGGGCGAGGCCTGCCCGCTGCTGGGCAGCCCGGGCGCTGGGCGAAATAGCGTCCTCTTCCTCCCCGTGGACCCCGAGGACTCGCCCCTTGGCAGCAGCACCCCCATGGCGTCTCCTGACCTCCTTCCAGAGGACGTGAGGGAGCACCTCGAAGGCTTGATGCTCTCGCTCTTCGAGCAGAGTCTGAGCTGCCAGGCCCAGGGGGGCTGCAGTAGACCCGCCATGGTCCTCACAGACCCACACACGCCCTACGAGGAGGAGCAGCGGCAGTCAGTGCAGTCTGACCAGGGCTACATCTCCAGGAGCTCCCCGCAGCCCCCCGAGGGACTCACGGAAATGGAGGAAGAGGAGGAAGAGGAGCAGGACCCAGGGAAGCCGGCCCTGCCACTCTCTCCCGAGGACCTGGAGAGCCTGAGGAGCCTCCAGCGGCAGCTGCTTTTCCGCCAGCTGCAGAAGAACTCGGGCTGGGACACGATGGGGTCAGAGTCAGAGGGGCCCAGTGCA | |

Table S2. The list of 3′-UTR mutated sequence of the IL17RA.

| plasmid | PGMLV-CMV-H_IL17RA(c.A2319C)-3×Flag-EF1-ZsGreen1-T2A-Puro |
| --- | --- |
| GCCACCATGGGGGCCGCACGCAGCCCGCCGTCCGCTGTCCCGGGGCCCCTGCTGGGGCTGCTCCTGCTGCTCCTGGGCGTGCTGGCCCCGGGTGGCGCCTCCCTGCGACTCCTGGACCACCGGGCGCTGGTCTGCTCCCAGCCGGGGCTAAACTGCACGGTCAAGAATAGTACCTGCCTGGATGACAGCTGGATTCACCCTCGAAACCTGACCCCCTCCTCCCCAAAGGACCTGCAGATCCAGCTGCACTTTGCCCACACCCAACAAGGAGACCTGTTCCCCGTGGCTCACATCGAATGGACACTGCAGACAGACGCCAGCATCCTGTACCTCGAGGGTGCAGAGTTATCTGTCCTGCAGCTGAACACCAATGAACGTTTGTGCGTCAGGTTTGAGTTTCTGTCCAAACTGAGGCATCACCACAGGCGGTGGCGTTTTACCTTCAGCCACTTTGTGGTTGACCCTGACCAGGAATATGAGGTGACCGTTCACCACCTGCCCAAGCCCATCCCTGATGGGGACCCAAACCACCAGTCCAAGAATTTCCTTGTGCCTGACTGTGAGCACGCCAGGATGAAGGTAACCACGCCATGCATGAGCTCAGGCAGCCTGTGGGACCCCAACATCACCGTGGAGACCCTGGAGGCCCACCAGCTGCGTGTGAGCTTCACCCTGTGGAACGAATCTACCCATTACCAGATCCTGCTGACCAGTTTTCCGCACATGGAGAACCACAGTTGCTTTGAGCACATGCACCACATACCTGCGCCCAGACCAGAAGAGTTCCACCAGCGATCCAACGTCACACTCACTCTACGCAACCTTAAAGGGTGCTGTCGCCACCAAGTGCAGATCCAGCCCTTCTTCAGCAGCTGCCTCAATGACTGCCTCAGACACTCCGCGACTGTTTCCTGCCCAGAAATGCCAGACACTCCAGAACCAATTCCGGGGCCTGGAAGTGAAAAATACAGTGATGACACCAAATACACCGATGGCCTGCCTGCGGCTGACCTGATCCCCCCACCGCTGAAGCCCAGGAAGGTCTGGATCATCTACTCAGCCGACCACCCCCTCTACGTGGACGTGGTCCTGAAATTCGCCCAGTTCCTGCTCACCGCCTGCGGCACGGAAGTGGCCCTGGACCTGCTGGAAGAGCAGGCCATCTCGGAGGCAGGAGTCATGACCTGGGTGGGCCGTCAGAAGCAGGAGATGGTGGAGAGCAACTCTAAGATCATCGTCCTGTGCTCCCGCGGCACGCGCGCCAAGTGGCAGGCGCTCCTGGGCCGGGGGGCGCCTGTGCGGCTGCGCTGCGACCACGGAAAGCCCGTGGGGGACCTGTTCACTGCAGCCATGAACATGATCCTCCCGGACTTCAAGAGGCCAGCCTGCTTCGGCACCTACGTAGTCTGCTACTTCAGCGAGGTCAGCTGTGACGGCGACGTCCCCGACCTGTTCGGCGCGGCGCCGCGGTACCCGCTCATGGACAGGTTCGAGGAGGTGTACTTCCGCATCCAGGACCTGGAGATGTTCCAGCCGGGCCGCATGCACCGCGTAGGGGAGCTGTCGGGGGACAACTACCTGCGGAGCCCGGGCGGCAGGCAGCTCCGCGCCGCCCTGGACAGGTTCCGGGACTGGCAGGTCCGCTGTCCCGACTGGTTCGAATGTGAGAACCTCTACTCAGCAGATGACCAGGATGCCCCGTCCCTGGACGAAGAGGTGTTTGAGGAGCCACTGCTGCCTCCGGGAACCGGCATCGTGAAGCGGGCGCCCCTGGTGCGCGAGCCTGGCTCCCAGGCCTGCCTGGCCATAGACCCGCTGGTCGGGGAGGAAGGAGGAGCAGCAGTGGCAAAGCTGGAACCTCACCTGCAGCCCCGGGGTCAGCCAGCGCCGCAGCCCCTCCACACCCTGGTGCTCGCCGCAGAGGAGGGGGCCCTGGTGGCCGCGGTGGAGCCTGGGCCCCTGGCTGACGGTGCCGCAGTCCGGCTGGCACTGGCGGGGGAGGGCGAGGCCTGCCCGCTGCTGGGCAGCCCGGGCGCTGGGCGAAATAGCGTCCTCTTCCTCCCCGTGGACCCCGAGGACTCGCCCCTTGGCAGCAGCACCCCCATGGCGTCTCCTGACCTCCTTCCAGAGGACGTGAGGGAGCACCTCGAAGGCTTGATGCTCTCGCTCTTCGAGCAGAGTCTGAGCTGCCAGGCCCAGGGGGGCTGCAGTAGACCCGCCATGGTCCTCACAGACCCACACACGCCCTACGAGGAGGAGCAGCGGCAGTCAGTGCAGTCTGACCAGGGCTACATCTCCAGGAGCTCCCCGCAGCCCCCCGAGGGCCTCACGGAAATGGAGGAAGAGGAGGAAGAGGAGCAGGACCCAGGGAAGCCGGCCCTGCCACTCTCTCCCGAGGACCTGGAGAGCCTGAGGAGCCTCCAGCGGCAGCTGCTTTTCCGCCAGCTGCAGAAGAACTCGGGCTGGGACACGATGGGGTCAGAGTCAGAGGGGCCCAGTGCA | |
| Mutation site: ACTCACGGAA->CCTCACGGAA | |

Table S3. The list of antibodies.

| Antibody | Company | Catalogue Number |  |
| --- | --- | --- | --- |
| IL17RA | Abcam | ab180904 |  |
| FTO | Abcam | ab126605 | For human samples |
| FTO | Abcam | ab92821 | For mouse samples |
| ALKBH5 | Abcam | ab195377 |  |
| METTL3 | Zen bio | 382974 |  |
| METTL14 | Sigma | HPA038002 |  |
| m6A | Synaptic Systems | 202003 |  |
| Beta-Actin | Proteintech | 66009-1 |  |
| Goat Anti-Mouse | LI-COR | 926-68070 |  |
| Goat Anti-Rabbit | LI-COR | 926-32211 |  |

Table S4. The list of primers.

| Official Gene Symbol | Gene ID | forward 5'-3' | reverse 5'-3' |
| --- | --- | --- | --- |
| homoFTO | 79068 | TGGGTTCATCCTACAACGG | CCTCTTCAGGGCCTTCAC |
| homoIL17RA | 23765 | AGACACTCCAGAACCAATTCC | TCTTAGAGTTGCTCTCCACCA |
| homoIL17RA-3UTR | 23765 | GCTCCATTATTCGTTCATT | CACTAGCGGTTAAGTTCCT |
| musFTO | 26383 | CTGGCATCACGATGAGAACC | CAGGAAAGGAAAGGACACCC |
| musIL17RA-3UTR | 16172 | ACTTGGGAACTGAGACTTG | GTCGGGTTATCAGGGAAA |

Table S5. Estimation or NAFLD.

NAFLD Activity Score (NAS)

Pathological appearance Assessment Score (NAS)

Hepatocyte ballooning None 0

Few ballooning cells 1

Many 2

Lobular inflammation: No foci 0

Overall assessment of all <2 foci per 200x field 1

Inflammation foci 2-4 foci per 200x field 2

>4 foci per 200x field 3

Steatosis: Low-to medi- <5% 0

um-power evaluation of 5%-33% 1

parenchymal involvement 33%-66% 2

by steatosis >66% 3

Pathological diagnosis Total score(NAS)

Probable or definite NASH >5

Uncertain 3-4

Not NASH <2
